# Case report: living donor liver transplantation for giant hepatic hemangioma using a right lobe graft without the middle hepatic vein

**DOI:** 10.1186/1477-7819-12-83

**Published:** 2014-04-04

**Authors:** Lin Zhong, Tong-Yi Men, Gao-di Yang, Yan Gu, Guoqing Chen, Tong-Hai Xing, Jun-Wei Fan, Zhi-Hai Peng

**Affiliations:** 1 Department of General Surgery, Shanghai First People Hospital, Medical School of Shanghai Jiaotong University, 85 Wu Jing Road, 200080 Shanghai, People’s Republic of China; 2 Department of Surgery, Shandong Province Qianfoshan Hospital, Shandong University, Jinan 250014, People’s Republic of China; 3 Shandong Academy of Medical Science, Jinan 250062, People’s Republic of China

**Keywords:** Giant hepatic hemangioma, Living donor liver transplantation

## Abstract

Hepatic hemangioma patients with Kasabach-Merritt syndrome have reportedly been cured by liver transplantation. However, liver transplantation as a potential cure for a stable patient without Kasabach-Merritt syndrome remains debatable. We report the case of a 27-year-old female patient with a giant hepatic hemangioma. The hemangioma measured 50 × 40 × 25 cm in size and weighed 15 kg, which is the largest and heaviest hemangioma reported in the literature. The patient showed jaundice, ascites, anemia, and appetite loss; but no disseminated intravascular coagulation was observed through laboratory findings. We successfully operated using a right lobe graft without the middle hepatic vein from a 55-year-old donor. At the long-term follow-up, the patient experienced two acute rejections, which were confirmed by biopsy. However, the patient still survives with good graft function after 50 months.

## Background

Hepatic hemangiomas are the most commonly occurring benign tumors of the liver. These tumors often remain asymptomatic when their diameter is smaller than 4 cm [1]. The available treatment options include radiation, interferon therapy, and embolization [1]. Surgical resection is the most common treatment modality [2]. A stable hemangioma has a rupture rate of 1% to 4% and a fatality rate of 60% to 75% after rupture. Kasabach-Merritt syndrome is characterized by the occurrence of disseminated intravascular coagulation that results from a hepatic hemangioma, and has a fatality rate of 10% to 37% that rises to 80% in the first year [3]. It has been reported that hepatic hemangioma patients with Kasabach-Merritt syndrome have been cured by liver transplantation [4,5].

We report the case of a 27-year-old female patient who had a giant hepatic hemangioma measuring 50 × 40 × 25 cm in size and weighing 15 kg; this is the largest and heaviest hemangioma reported in the literature [6,7]. Patients with a high Model for End-stage Liver Disease (MELD) score caused by Kasabach-Merritt syndrome or with a ruptured tumor are eligible for receiving organs from the cadaveric donor pool. However, the progress of a stable hemangioma can cause abnormal coagulation mechanisms or hemangioma rupture, which results in the loss of opportunity for operation or increased operation risk. Living donor liver transplantation is therefore the best option in these cases. This surgical method thus solves the problem of the source of the graft, and possesses a high level of safety. The implementation of this method may determine the progress of the operation and the nature of the outcome. In this case, the patient received a living donor liver transplantation for a giant hepatic hemangioma using a right lobe graft without the middle hepatic vein (MHV) [8].

## Case presentation

**Figure 1:**
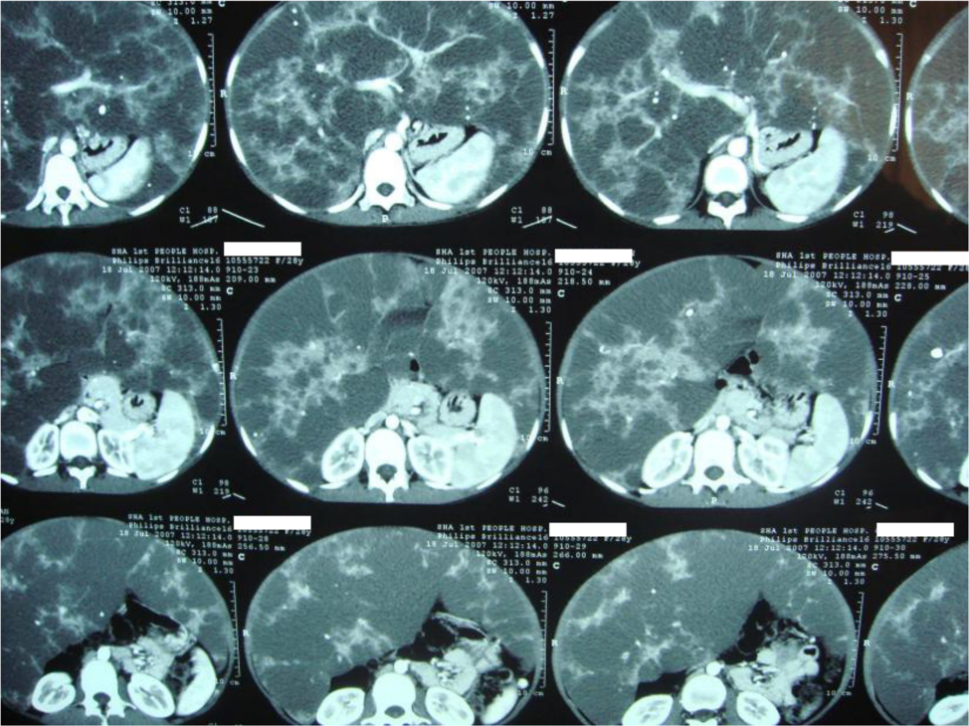
CT scan shows the giant hepatic hemangioma occupying the entire abdominal cavity.

**Figure 2:**
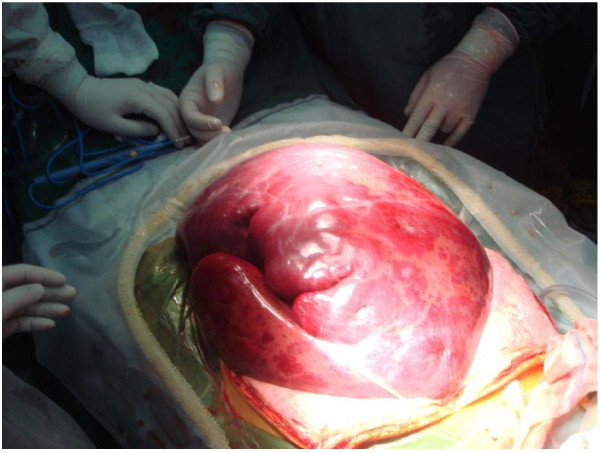
The giant hemangioma during surgery, approximately 50 × 40 × 25 cm in size.

A 27-year-old female patient, who experienced upper abdominal pain and progressive hepatoma for 4 years, was diagnosed with a giant hepatic hemangioma. The patient showed mild jaundice, abdominal fullness, and abdominal varicose veins on presentation. Laboratory examination showed a hemoglobin level of 81 g/L and a platelet count of 95 × 10^9^/L. Blood biochemistry showed total bilirubin concentration of 1.34 mg/dL, alanine aminotransferase concentration of 10 U/L, and creatinine level of 0.44 mg/dL. The coagulation tests showed a prothrombin time (PT) of 15.4 seconds, activated partial thromboplastin time of 35 seconds, fibrinogen concentration of 4.2 g/L, and fibrinogen degradation product tests were negative. An abdominal CT scan indicated the presence of low-density areas in both the liver lobes, occupying the entire abdominal cavity without rupture (Figure 1). The recipient was further examined and this revealed a hemangioma occupying the entire abdominal cavity, measuring approximately 50 × 40 × 25 cm in size (Figure 2).

**Figure 3:**
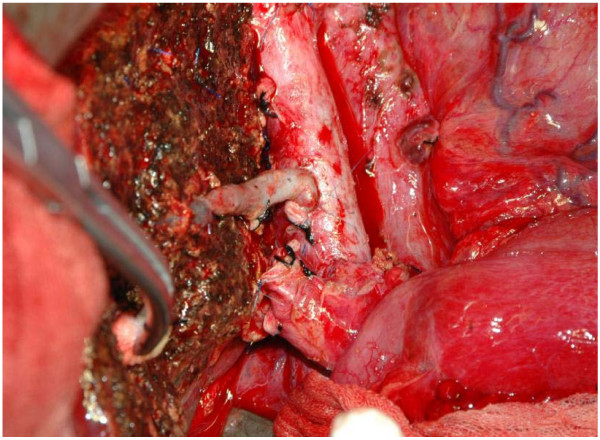
The donor’s V8 segment vein bridging to the inferior vena cava with good blood reflux.

**Figure 4:**
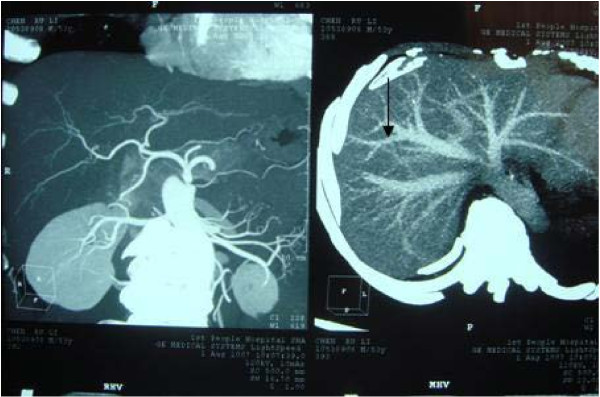
The donor’s hepatic artery and hepatic vein; the arrow shows the V8 segment vein.

Surgery was performed on September 1, 2007. The donor was the patient’s 55-year-old father. Preoperative evaluation showed that the graft-to-recipient weight ratio (GRWR) was 1.46% and the remnant liver volume (RLV) of the donor was 47%. During surgery, the liver was not mobilized and rotated to prevent the risk of hemangioma rupture. We ligated the portal canal and blocked the upper and lower blood supply to the liver, and then resected the recipient’s liver and simultaneously preserved the donor’s inferior vena cava. A hepatic occlusion clamp was used to control the blood flow to the liver from the inferior vena cava. End-to-end anastomosis was performed in order to join the recipient’s right hepatic vein and the donor’s right hepatic vein. The diameter of the V8 segment vein was 0.3 cm. We dealt with the inferior mesenteric vein by bridging the inferior vena cava to avoid congestion of the V8 segment vein (Figures 3 and 4). End-to-end anastomosis was also performed between the donor and recipient’s right branch of the portal vein, as well as for the donor’s and recipient’s common hepatic veins. The hepatic artery, portal vein, and bile duct was resected using the anterior approach. The portal vein was cut using the 55-mm cutting stapler (Johnson & Johnson) to avoid the effects of using a surgical clamp on the portal vein. *In situ* incision of the coronary ligament and deltoid ligament was performed to separate the hepatogastric ligament for the left side of the liver. Finally, 3 to 5 hepatic segment veins were ligated and incised along the hepatic inferior vena cava in the centripetal direction and the diseased liver was resected without moving the right liver lobe with retention of the retro-hepatic inferior vena cava.

**Figure 5:**
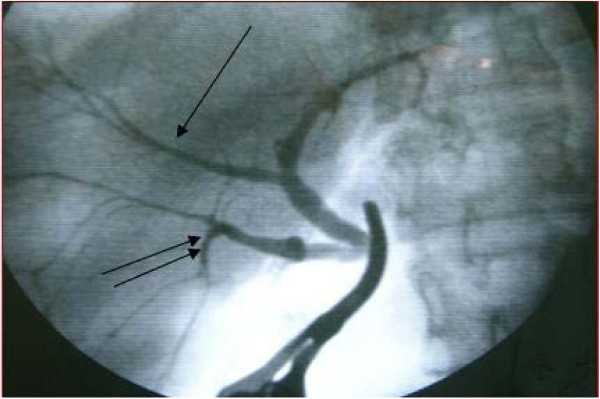
**Variation observed in the bile duct.** The right anterior branch imported into left hepatic duct. Right anterior branch and posterior branch were reconstructed into a single orifice that was anastomosed to the common hepatic duct of the recipient.

A variation in the donor’s bile duct was observed and dealt with surgically. The right anterior branch was connected to the left hepatic duct, which was clearly visible on bile duct radiography of the graft (Figure 5). The right anterior branch and the posterior branch were anastomosed to a single orifice.

The surgery was performed successfully without the transfusion of any blood products. The donor’s surgical time was approximately 3 hours and 10 minutes (the time spent on hepatectomy was 2 hours, excluding the initial abdominal entry and subsequent closure) and that of the recipient was approximately 4 and a half hours. The patient recovered gradually after operation without experiencing any infection or any vascular or biliary complications. She was discharged 20 days after surgery.

Postoperative pathology showed that the surface of the removed liver was an abnormal color ranging from gray-red to gray-yellow. A cross-sectional sample that was obtained was also gray-yellow in color. The entire liver comprised of tumorous tissue, with little normal hepatic tissue remaining. A giant hepatic cavernous hemangioma was diagnosed. The immunity class showed CD34 (+).

A liver biopsy 12 months after the operation confirmed an acute rejection reaction with a Buffer score of 9, but the patient recovered with methylprednisolone treatment. The second episode of rejection occurred at 17 months with a Buffer score of 7 and was treated by increasing the tacrolimus dosage and concentration. The patient currently remains healthy, and the graft is fully functional.

## Discussion

Hepatic hemangiomas are the most common hepatic tumors. The indications for liver transplantation in cases of hepatic hemangioma include acute and chronic hepatic failure, such as occurrence of Kasabach-Merritt syndrome, giant hepatic hemangioma affecting the normal liver tissue eventually causing liver dysfunction, and a life-threatening giant hepatic hemangioma that cannot be resected [9,10]. In this case, the patient’s hemangioma was very large as previously measured and described, but the patient was stable without Kasabach-Merritt syndrome or liver dysfunction. She did experience abdominal symptoms and showed an extended PT, as well as decreased platelet and hemoglobin levels. The risk of hemangioma rupture was present. If the patient was to wait for a rupture or a high MELD score in order to be eligible for receiving an organ from the cadaveric donor pool, the opportunity for surgery would have been lost or the transplantation risk would have been higher than before. Therefore, we decided to perform liver transplantation using a right lobe graft from the patient’s father, and thus, avoided the risk associated with an emergency surgery, ensuring increased safety associated with such a surgery.

It has been reported that reconstruction is indicated when the V8 diameter is greater than 5 mm [11]. However, in our experience, any blood vessels with a diameter greater than 2 mm should be reconstructed. In this particular case, even though the GRWR was 1.46%, the size of the donor liver was taken into consideration, and pre-operative tests have shown that the V8 accounted for 15% of the blood reflow in the donor liver. The immediate post-operative blood flow was greater than 260 mL/min and the venous pressure was increased. These factors increased the risk for small-for-size liver syndrome. From our observations, patients with GRWR >1.0 are also at higher risk of developing small-for-size liver syndrome due to increased venous pressure. Further, when the portal vein pressure is greater than 30 mmHg and the right half of the liver, excluding the hepatic vein, is used, congestion around the midline of the liver near V5 and V8 can result if the hepatic vein is not reconstructed. Therefore, reconstruction of the hepatic vein can decrease the amount of congestion. Finally, due to the liver donor being above 55 years old, there was mild hepatic lipidosis and fibrosis in the donor liver, decreasing the ability of the donor liver to recover post-transplantation. Reconstructing the V8 can aid recovery of the hepatocytes, thus preventing small-for-size liver syndrome. Preoperative evaluation in cases of living donor liver transplantation is important. In general, a GRWR of >0.8% and a donor RLV of >40% are two important parameters [4]. The values of these two vital parameters in this case were 1.46% and 47%, respectively, thus guaranteeing the safety of both donor and recipient.

Outflow impairment of the hepatic vein after surgical transplantation, also known as small-for-size syndrome, is a common problem [12]. We used the right lobe graft without the MHV as the donor organ. The diameter of the V8 segment vein was 0.3 cm, which could drain sufficient blood to avoid the risk of small-for-size syndrome. As mentioned previously, a variation in the donor’s bile duct was found and dealt with by connecting the right anterior branch to the left hepatic duct, which was clearly visible on bile duct radiography of the graft. The reported rate of biliary complications in patients with such variations ranges from 25% to 35%, and is high in living donor liver transplant recipients [13-15]. To address this problem, we reconstructed the right anterior branch and posterior branch into a single orifice that was anastomosed to the common hepatic duct of the recipient.

**Figure 6:**
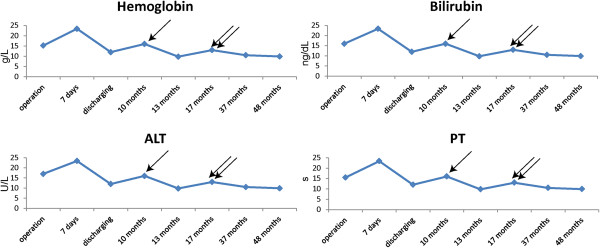
**Showing the four important indices of the patient.** Two acute rejection reactions occurred at 12 months and 17 months, which were confirmed by biopsy. The single arrow indicates the first rejection and the double-arrows indicate the second. The two peaks from the table reflect these two episodes.

There are few long-term reports on the outcome of liver transplantation for a giant hepatic hemangioma. We have continued follow-ups with this patient for 50 months. The graft functions well, without biliary complications or hemangioma recurrence. As described earlier, two occasions of acute rejection were diagnosed and treated successfully (Figure 6).

It is thought that the age of the donor and the risk of rejection might be related. According to previous reports, an older donor presents a higher rejection risk. However, other researchers believe that there is no apparent difference in the rejection rate and survival rate of those receiving grafts from older donors [16]. As a result, the safety and rejection risk associated with older donors requires more research and discussion.

## Conclusions

In summary, we believe that liver transplantation should be performed early for an unresected giant diffuse hemangioma. In our experience, a good long-term outcome can be expected following liver transplantation using a right lobe graft without the MHV.

## Consent

Written informed consent was obtained from the patient for publication of this Case Report and any accompanying images. A copy of the written consent is available for review by the Editor-in-Chief of this journal.

## Abbreviations

GRWR: Graft-to-recipient weight ratio; MELD: Model for End-stage Liver Disease; MHV: Middle hepatic vein; PT: Prothrombin time; RLV: Remnant liver volume.

## Competing interests

The authors declare that they have no competing interests.

## Authors’ contributions

LZ and ZHP performed the surgery; LZ, TYM and GDY wrote the manuscript; GY helped collecting references; GQC, THX and JWF also attended surgery and helped post-operation management. All authors read and approved the final manuscript.
